# Feature Preserving Autofocus Algorithm for Phase Error Correction of SAR Images

**DOI:** 10.3390/s21072370

**Published:** 2021-03-29

**Authors:** Haemin Lee, Chang-Sik Jung, Ki-Wan Kim

**Affiliations:** Agency for Defense Development, Yuseong P.O. Box 35, Daejeon 34186, Korea; csjung@add.re.kr (C.-S.J.); kiwankim@add.re.kr (K.-W.K.)

**Keywords:** autofocus, feature preserving regularization, phase errors, synthetic aperture radar (SAR)

## Abstract

Autofocus is an essential technique for airborne synthetic aperture radar (SAR) imaging to correct phase errors mainly due to unexpected motion error. There are several well-known conventional autofocus methods such as phase gradient autofocus (PGA) and minimum entropy (ME). Although these methods are still widely used for various SAR applications, each method has drawbacks such as limited bandwidth of estimation, low convergence rate, huge computation burden, etc. In this paper, feature preserving autofocus (FPA) algorithm is newly proposed. The algorithm is based on the minimization of the cost function containing a regularization term. The algorithm is designed for postprocessing purpose, which is different from the existing regularization-based algorithms such as sparsity-driven autofocus (SDA). This difference makes the proposed method far more straightforward and efficient than those existing algorithms. The experimental results show that the proposed algorithm achieves better performance, convergence, and robustness than the existing postprocessing autofocus algorithms.

## 1. Introduction

Synthetic aperture radar (SAR) can create high cross-range resolution images through coherent processing of the returned pulses received at different antenna position due to a moving platform, which enables provision for the effect of a large virtual aperture size [1,2,3]. To achieve full performance of SAR, the exact flight trajectory of the platform should be provided to the signal processor for proper motion compensation. Especially in the case of airborne SAR, the effect of the unexpected platform motion due to the factors such as wind gusts and aircraft vibrations should be compensated before SAR processing. These compensations for airborne SAR are usually performed by using the measured navigation data such as the output of global positioning system (GPS), inertial navigation system (INS), or embedded GPS/INS (EGI) [4]. However, these data also contain the measurement errors due to the inaccuracy of the navigation sensors, and these residual errors cause the phase errors of SAR data, which degrades the quality of the SAR image. There are several methods to solve this kind of image quality degradation, which are usually called autofocus. 

Phase gradient autofocus (PGA) is one of the most widely used algorithms to estimate the phase error [5]. The method assumes all the complex reflectivity in the image windowed with appropriate window size, except the center-shifted point target at each range bin, are distributed as zero-mean Gaussian random noises. These assumptions limit the performance of the algorithm in spite of its robustness and fast convergence. The point target and random noise assumption are inappropriate to the scene that contains dominant targets close to each other in the azimuth direction. Quality phase gradient autofocus (QPGA) and generalized phase gradient autofocus (GPGA) are proposed to alleviate this problem [6,7]. However, both QPGA and GPGA use the limited window size, which also limits the bandwidth of the estimated phase error. Therefore, these PGA-based methods are not suitable for estimating the phase error containing the high frequency components such as the error generated by the GPS measurement update in EGI.

Optimization of sharpness metrics is also a well-known autofocus technique [8,9]. These kinds of methods are used to optimize the cost function, which enables the scene to be well focused. One of the most commonly used cost functions is entropy, and such methods are often called the minimum entropy (ME) method [10,11,12,13,14,15]. ME-based methods are not restricted by the assumptions in PGA, so there is no limitation on the bandwidth of estimation. There are various ways to achieve the optimization because these methods have no closed-form solutions [8]. One way is to model the estimated error as a polynomial and adjust the coefficients to minimize the entropy of the image [10]. Although this method achieves appropriate performance, the higher order components of the phase errors are difficult to estimate because of the limited order of the estimates, which is similar to the bandwidth issue in PGA. Even though the order of the polynomial can be adaptively increased, it would be computationally intensive. The optimization method based on the trial and error [11] also experiences similar or a more extensive computational problem. 

The most widely used ME methods can be divided into two categories. The first is based on gradient searching. One of the well-known methods in this category is the monotonic iterative algorithm (MIA) [12], which minimizes well-defined surrogate functions instead of the entropy. An algorithm based on Newton’s method to obtain the phase estimate, which makes the gradient of entropy zero, also provides reasonable performance [13]. These methods can be carried out through the fast Fourier transform (FFT), which enables the fast phase estimation. Another category is based on the fixed-point iteration [14,15]. In these methods, the fixed-point iteration is used to solve the implicit equation of the phase, which makes the derivative of the entropy zero. Every iteration can also be conducted through FFT. The algorithms in these two categories show similar estimation accuracy and convergence.

Regularization-based autofocus techniques are also proposed by some authors [16,17,18,19,20,21,22], which are methods that are quite different from postprocessing autofocus such as PGA and ME. These methods are based on a regularized reconstruction of the SAR images, which are often called compressive sensing [22], sparsity-driven imaging [23], or feature-enhanced SAR imaging [24,25,26]. Unlike the conventional SAR image formation such as the polar format algorithm (PFA) [1], regularization-based imaging methods have advantages such as high resolution, which is not limited by the SAR system bandwidth, and suppression of the artifact owing to speckle and side-lobe, even if the datasets are nonuniform and undersampled. Sparsity-driven autofocus (SDA), one of the well-known autofocus methods in this area, is to minimize the cost function, composed of a fidelity and regularization term, jointly with the estimate of image and the phase error [16]. SDA achieves quite accurate phase estimation with preservation of the advantages of regularized reconstruction. Similar approaches have been proposed such as optimization of cost function, including total variation [17] and modified Tikhonov regularization-based autofocus [18,19] to improve the performance and reduce the computational burden. Although the regularization-based methods achieve high quality SAR images, these are hard to utilize for the SAR mission, which requires on-board processing and a large scene size. The reason is that these methods perform not only the estimation of phase error but also the reconstruction of the image simultaneously, which requires huge computation power. The large computation burden of regularized reconstruction is one of the reasons why PFA is still widely utilized for on-board SAR image formation. If the SAR system is well designed to meet the performance requirements, the only concern for image degradation is the phase error, not the sparse sampling.

In this paper, we propose a new postprocessing autofocus algorithm for phase-corrupted images. The algorithm is designed to minimize the cost function containing regularization term based on l_1_-norm, which is similar to regularization-based autofocus such as SDA. However, the proposed algorithm deals with the processed complex image corrupted by the phase error, whereas SDA deals with returned pulse data before processing. This difference makes the proposed algorithm straightforward and requires only simple calculation for FFT and soft-threshold. The equation for minimization of the cost function is carried out through an indirect optimization manner, and fixed-point iteration is used to obtain the optimal solution.

The rest of this paper is outlined as follows. In Section 2, the fundamental background for the proposed algorithm, such as iterative shrinkage thresholding algorithm (ISTA) and denoising [27], are explained. In Section 3, we define the cost function for the proposed method and present the iterative algorithm to achieve the minimization. Then, we demonstrate the performance, convergence, and robustness of the proposed algorithm and compare them with those of the existing autofocus methods such as PGA, GPGA, and ME through experimental results in Section 4. Finally, the conclusions are presented in Section 5.

## 2. Fundamental Background

### 2.1. Phase Error Correction in SAR Images

The typical SAR image formations are achieved through the two-dimensional inverse discrete Fourier transform (DFT) of the motion compensated echo signal formatted to Cartesian coordinates. If we define the range-compressed, motion compensated echo signal as G(n, m), where n and m are the indices of range cells of the scene and received pulses, respectively, the complex image g(n, k) is formulated as

(1) $$g(n,\;k)=\sum_{m=0}^{M-1}G(n,\;m)\exp\left(j\frac{2\pi }{M}km\right)$$

where k is the indices of azimuth cells of the scene, M is the number of received pulses, and $j^{2}=-1$. The residual phase errors after the motion compensation in the SAR image are mainly due to the errors in the navigation data, and these errors corrupt each returned pulse signal phase. Therefore, most of the phase distortions are in azimuth direction, and the phase-corrected complex image can be formulated as

(2) $$\hat{g}(n,\;k)=\sum_{m=0}^{M-1}G(n,\;m)\exp\left[j\varphi (m)\right]\exp\left(j\frac{2\pi }{M}km\right)$$

where φ(m) is the estimated residual phase error. If we can obtain an accurate estimation of the phase error, the corrected image is also obtained through (2), which can be carried out through FFT.

### 2.2. Phase Adjustment for Desired Image

If we have the desired complex image and want to obtain the phase adjustment for minimizing the error between the desired and phase adjusted image, we can formulate the following cost function:

(3) J=12∑k=0M−1∑n=0N−1|g^(n, k)−h(n, k)|2=Re[∑k=0M−1∑n=0N−1g^(n, k)h∗(n,k)] +12∑k=0M−1∑n=0N−1|g^(n, k)|2+12∑k=0M−1∑n=0N−1|h(n, k)|2

where h(n, k) is the desired complex image and N is the number of range cells of the scene; ${\left(\cdot \right)}^{*}$ and Re(⋅) denotes complex conjugate and the real part of the complex number, respectively. We assumed that the number of azimuth cells of the scene is equal to M. Let the derivative with respect to φ(m) be zero to minimize the cost function in (3):

(4) ∂J∂φ(m)=∂∂φ(m){Re[∑k=0M−1∑n=0N−1g^(n, k)h∗(n,k)]}         =Re[∑k=0M−1∑n=0N−1∂g^(n, k)∂φ(m)h∗(n,k)]=0

The second and third additive terms in (3) are ignored because the phase does not have an effect to the variation of the total intensity. The derivative of $\hat{g}(n,\;k)$ with respect to φ(m) is derived from (2) as given in the following equation:

(5) $$\frac{\partial \hat{g}(n,\;k)}{\partial \varphi (m)}=jG(n,\;m)\exp\left[j\varphi (m)\right]\exp\left(j\frac{2\pi }{M}km\right)$$

Then, (4) becomes

(6) ∂J∂φ(m) =Im{exp[jφ(m)]∑n=0N−1G(n, m)H∗(n, m)}=0

where Im(⋅) denotes the imaginary part of the complex number and H(n, m) is defined as

(7) $$H(n,\;m)=\sum_{k=0}^{M-1}h(n,\;k)\exp\left(-j\frac{2\pi }{M}km\right)$$

H(n, m) in (7) can be computed through FFT. From (6), the sufficient solution for φ(m) is obtained from the following equation:

(8) $$\varphi (m)=∠\left[\sum_{n=0}^{N-1}G^{*}(n,\;m)H(n,\;m)\right]$$

where ∠(⋅) denotes the phase of the complex number. The result in (8) will be used in Section 3 to explain the proposed method. 

### 2.3. Brief Review of Regularized Reconstruction

SAR image formation is basically solving an inverse problem to obtain the image from the returned signal. If we know the exact SAR observation model, SAR imaging is identical to solving the following equation with respect to x [25]:

(9) y=Cx+v

where x, y, and v comprise an MN×1 vector, which is the column stacked version of the complex image, returned echo, and measurement noise, respectively, and C is the discretized observation kernel. The following cost function is usually used to solve the inverse problem in (9) [25]:

(10) $$J=\frac{1}{2}{\left\|y-Cx\right\|}_{2}^{2}+\lambda {\left\|x\right\|}_{p}^{p}$$

where ${\left\|\cdot \right\|}_{p}$ denotes the l_p_-norm and λ is a weighting parameter for regularization term. The first term represents the fidelity term for preserving the dependence on the observation model and the second term is the regularization term for enhancement of the feature of the image. The typical choice for p is 1 [26]. The optimization can be achieved through two ways, one is the gradient descent algorithm [26] and the other is the iterative shrinkage thresholding algorithm (ISTA) [28]. For p=1, ISTA is represented as

(11) $$x_{i+1}=S_{\lambda }\left(x_{i}-\alpha C^{H}(Cx_{i}-y)\right)$$

where α is a sufficiently small positive number, $x_{i}$ is estimate of x in i-th iteration, and $S_{\lambda }(\cdot )$ is a soft-thresholding function defined as

(12) $$\begin{array}{l} S_{\lambda }\left(x\right)=\exp\left[j(∠x)\right]{\left(\left|x\right|-\lambda \right)}_{+}\\ \;\;\;\left({\left(x\right)}_{+}=\left\{ \begin{array}{l} x\;\mathrm{if}\;x\geq 0\\ 0\;\mathrm{otherwise} \end{array}\right.\right) \end{array}$$

Iteration in (11) achieves the optimal image for (10) if α is smaller than the largest eigenvalue of $C^{H}C$. Unlike the inverse problem of (9) and (10), if we already have the complex image formatted by (1) and want to carry out the feature enhancement in postprocessing, x and y become the column stacked version of the complex image before and after the postprocessing, respectively, and kernel C becomes the identity matrix. Then, (10) is rewritten as

(13) $$\begin{array}{ll} J & =\frac{1}{2}{\left\|y-x\right\|}_{2}^{2}+\lambda {\left\|x\right\|}_{1}\\ & =\frac{1}{2}\sum_{k=0}^{M-1}\sum_{n=0}^{N-1}\left({\left|g(n,\;k)-\bar{g}(n,\;k)\right|}^{2}+\lambda \left|\bar{g}(n,\;k)\right|\right) \end{array}$$

where $\bar{g}(n,\;k)$ is the postprocessed complex image. Unlike the iterative solution in (11), the optimization of (13) can be achieved by the following the closed-form solution [27]:

(14) $$\bar{g}(n,\;k)=S_{\lambda }\left(g(n,\;k)\right)$$

If we approximate $\left|\bar{g}(n,\;k)\right|$ to be ${\left[{\bar{g}}^{*}(n,\;k)\bar{g}(n,\;k)+\varepsilon \right]}^{\frac{1}{2}}$ for a sufficiently small positive scalar ε, then it satisfies

(15) $${\frac{\partial J}{\partial \bar{g}(n,k)}|}_{\bar{g}(n,k)=S_{\lambda }\left(g(n,\;k)\right)}\approx 0$$

From (14), each element of $\bar{g}$ whose absolute value is smaller than λ is removed, and the absolute value of the other elements are decrease by λ. This so-called “denoising” [28] is an important procedure for the proposed algorithm described in the next section.

## 3. Proposed Method

In this section, we define a cost function and derive the equation for its optimality in an indirect optimization manner. We then propose an algorithm to obtain the solution for the equation.

### 3.1. Cost Function and Its Minimization

The phase-corrupted image, owing to the residual phase error, shows distorted features such as degraded resolution and unexpected side-lobes, and hence the goal for phase estimation can be interpreted as to remove these distortions. Therefore, the objective of our method is a phase adjustment to achieve the feature enhancement, which can be realized by concurrent optimization of (3) and (13), i.e., the following cost function:

(16) $$J\;(\bar{g},\;\;\varphi )=\frac{1}{2}\sum_{k=0}^{M-1}\sum_{n=0}^{N-1}\left({\left|\hat{g}(n,\;k)-\bar{g}(n,\;k)\right|}^{2}+\lambda \left|\bar{g}(n,\;k)\right|\right)$$

where $\hat{g}$ is the function of φ according to (2). The minimization of (16) enables obtaining the feature-enhanced image $\bar{g}$ and the phase adjustment φ to make the phase-corrected image close to $\bar{g}$. We can achieve the minimization through the following indirect optimization.

Let $\bar{g}$ be the solution of the necessary condition for the minimum of (16), i.e., $\partial J/\partial \bar{g}=0$; then, the solution is obtained from (14) as

(17) $$\bar{g}(n,\;k)=S_{\lambda }\left(\hat{g}(n,\;k)\right)$$

If (17) holds for every φ, then we can replace the cost function in (16) by the following indirect cost function:

(18) $$V(\;\varphi )=\frac{1}{2}\sum_{k=0}^{M-1}\sum_{n=0}^{N-1}\left({\left|\hat{g}(n,\;k)-S_{\lambda }\left(\hat{g}(n,\;k)\right)\right|}^{2}+\lambda \left|S_{\lambda }\left(\hat{g}(n,\;k)\right)\right|\right)$$

The derivative of (18) with respect to φ is

(19) $$\frac{\partial V(\varphi )}{\partial \varphi }=\frac{\partial J(\bar{g},\;\;\varphi )}{\partial \bar{g}}\frac{\partial \bar{g}}{\partial \varphi }+\frac{\partial J(\bar{g},\;\;\varphi )}{\partial \varphi }\;\approx \frac{\partial J(\bar{g},\;\;\varphi )}{\partial \varphi }$$

because $\partial J/\partial \bar{g}\approx 0$ for $\bar{g}$ in (17). Let (19) to be zero for minimization of V( φ), then the condition for optimality can be derived by using the result in (6) as given in the following equation:

(20) ∂J∂φ(m) =Im{exp[jφ(m)]∑n=0N−1G(n, m)G¯∗(n, m)}=0

where $\bar{G}(n,\;m)$ is defined as

(21) $$\begin{array}{l} \bar{G}(n,\;m)=\sum_{k=0}^{M-1}\bar{g}(n,\;k)\exp\left(-j\frac{2\pi }{M}km\right)\\ \;\;\;\;\;\;\;\;\;\;\;\;\;\;\;\;=\sum_{k=0}^{M-1}S_{\lambda }\left(\hat{g}(n,\;k)\right)\exp\left(-j\frac{2\pi }{M}km\right) \end{array}$$

The solution of (20) is the final phase error estimate of our proposed method. 

### 3.2. Algorithm

The sufficient condition for (20) can be written in a manner similar to that in (8):

(22) $$\varphi (m)=∠\left[\sum_{n=0}^{N-1}G^{*}(n,\;m)\bar{G}(n,\;m)\right]$$

**Figure 1:**
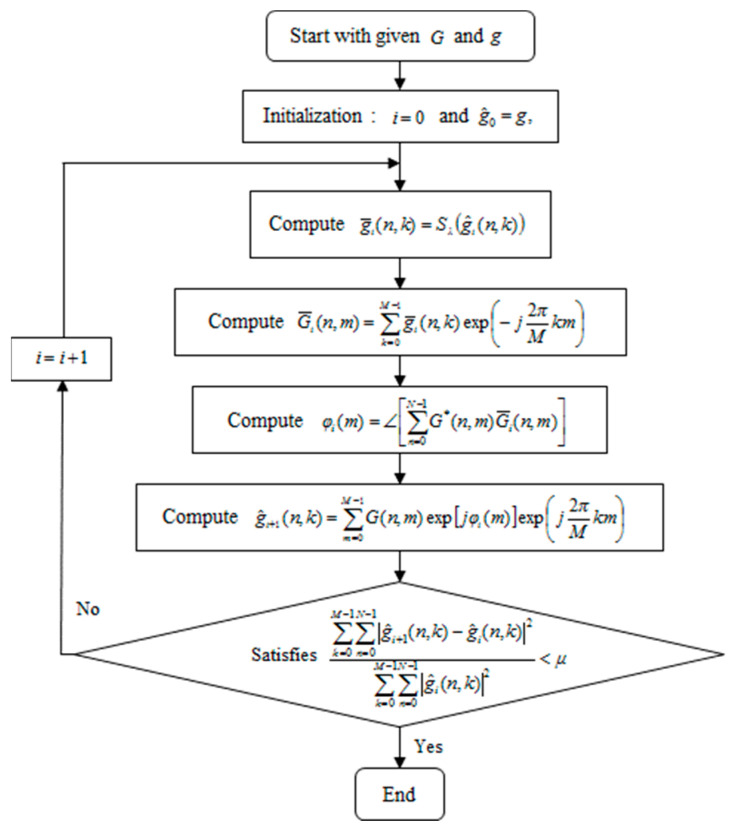
Flowchart of the proposed algorithm.

Equation (22) is not a closed-form solution of φ(m) unlike (8), because $\bar{G}(n,\;m)$ is still the function of φ(m). This implicit equation can be solved by the fixed-point iteration method, which has been applied to solve the similar problems for ME [13,14]. The flowchart of the proposed algorithm is represented in Figure 1. The computation of (2) and (21) in the algorithm can be carried out through FFT, which enables a fast iteration.

In every iteration, ${\bar{g}}_{i}$ takes the same role of the desired complex image, i.e., h in (7), which is the denoised image of ${\hat{g}}_{i}$. Because of the soft-thresholding function in (17), a few elements of ${\hat{g}}_{i}$ whose absolute value is larger than λ are preserved in ${\bar{g}}_{i}$ and the others are set to be zero. We call these preserved elements “features” in this paper. These remained features are the reference for the phase estimation in every iteration. In other words, the phase adjustment is achieved to fit the given image to the feature-preserved image. Therefore, we refer to the proposed algorithm as “feature preserving autofocus (FPA)” in this paper. Since the existing regularization-based autofocus algorithms such as SDA deal with the returned pulse data before processing, image formation algorithms such as the gradient descent algorithm and ISTA described in Section 2.3 are required for every iteration. Therefore, these algorithms require huge computation for a large scene size. Meanwhile, the proposed FPA requires only one soft-thresholding to obtain the reference image for phase adjustment. This simple iteration sequence makes the proposed algorithm more suitable for on-board SAR processing.

### 3.3. Selection of the Threshold

It is worth noting that a careful choice of the threshold, i.e., λ, is recommended for the proposed FPA. A choice of small λ enables the features to contain a sufficiently large number of scatterers, which achieves accurate estimation. However, if λ is too small, the remaining features would also contain the artifacts of the SAR image such as side-lobes even though their magnitude are reduced. Thus, it may cause low speed of convergence, and insufficient estimation accuracy if the artifacts still remain in the features at the final iteration. On the other hand, if λ is too large, some dominant scatterers for phase estimation would be removed from the features. In this case, the estimation is too concentrated in a few remained strong scatterers, which leads to local optimal estimation even if it enables to achieve faster convergence rate. 

To solve these tradeoffs, we propose varying the threshold to satisfy both fast convergence and optimal performance. The fast convergence can be achieved by setting a large λ at the initial iteration loop, and the accurate estimation can be carried out through gradually decreasing the value of λ for the rest of the iterations. For example, we suggest a simple asymptotically decreasing model for λ:

(23) $$\lambda_{i}=\alpha \lambda_{i-1}=\alpha^{i}\lambda_{0}$$

where α and $\lambda_{i}$ represent the positive forgetting factor less than 1 and the threshold for i-th iteration, respectively. The threshold for the initial loop, i.e., $\lambda_{0}$, would be a user-defined sufficiently large positive value, but small enough to achieve an appropriate number of features containing the main scatterers. There are various ways to select the main scatterers, such as the methods in [6,7,13]. Any of these methods would work to select the appropriate initial threshold. Alternatively, we observed that the proposed algorithm with an initial threshold slightly smaller than the maximum amplitude of the image, which enables the features to contain at least one scatterer, performs sufficient convergence and performance for every case in this paper. The performance comparison for constant and varying threshold is described in the next section. 

## 4. Experimental Results

In this section, some experimental results are demonstrated to verify the benefits of our proposed algorithm. In Section 4.1, we verify the performance and characteristics of the proposed method with a different threshold λ, and explain the tradeoff between the convergence and accuracy. Then, we compare the results for the constant threshold with those of the varying threshold to compromise the tradeoff, which is introduced in the previous section. In Section 4.2, we demonstrate the convergence and the performance of the proposed method with various types of the phase error, and compare them with those of the existing autofocus algorithms. PFA is utilized for SAR imaging to all the experimental results. The quantitative measures to verify the performance of the autofocus algorithm are defined as

(24) $$\begin{array}{l} IC\;(\hat{g})=\frac{\sqrt{E\left({\left[\left|\hat{g}(n,k)\right|-E\left\{\left|\hat{g}(n,k)\right|\right\}\right]}^{2}\right)}}{E\left[\left|\hat{g}(n,k)\right|\right]}\\ IE\;(\hat{g})=-\sum_{k=0}^{M-1}\sum_{n=0}^{N-1}\frac{{\left|\hat{g}(n,\;k)\right|}^{2}}{E\left[{\left|\hat{g}(n,\;k)\right|}^{2}\right]}\ln\left(\frac{{\left|\hat{g}(n,\;k)\right|}^{2}}{E\left[{\left|\hat{g}(n,\;k)\right|}^{2}\right]}\right) \end{array}$$

where E(⋅) represents the spatial mean operator, and $IC(\hat{g}\;)$ and $IE(\hat{g}\;)$ represent the contrast and entropy of the image $\hat{g}\;$, respectively. The initial image g is scaled to have a maximum magnitude of 1 for all the cases of the experiment, which makes the threshold λ for the soft-threshold function within 0 to 1. The constant μ for stop criteria is set to be 10^–4^ for all cases. 

### 4.1. Performance and Convergence of the Proposed Method

**Figure 2:**
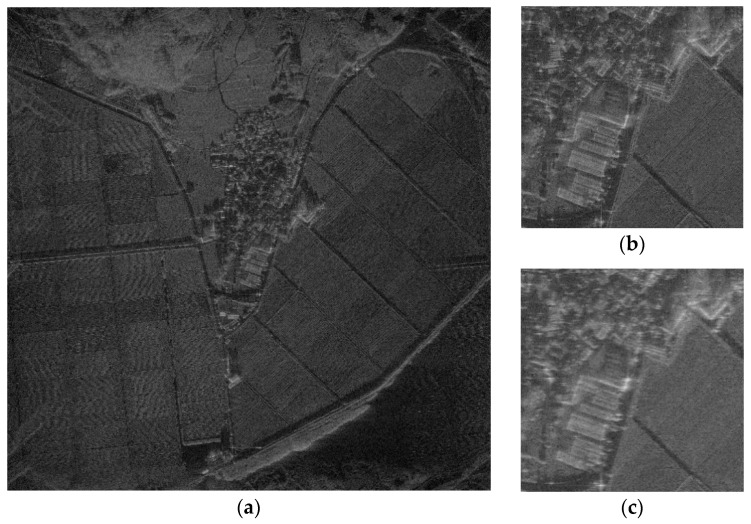
Synthetic aperture radar (SAR) image constructed by polar format algorithm (PFA). (**a**) Full size image. (**b**) Enlarged image without the phase error. (**c**) Enlarged image with the phase error.

**Figure 3:**
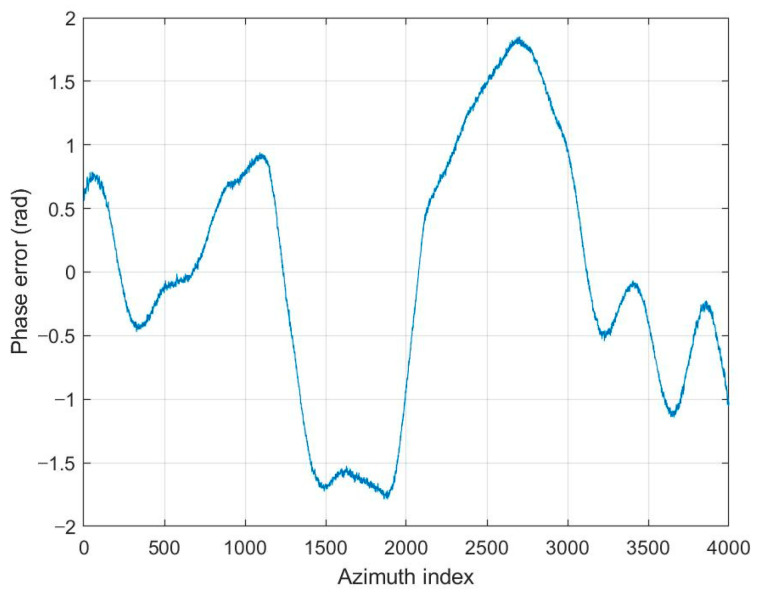
Phase error applied to the SAR image in Figure 2c.

**Figure 4:**
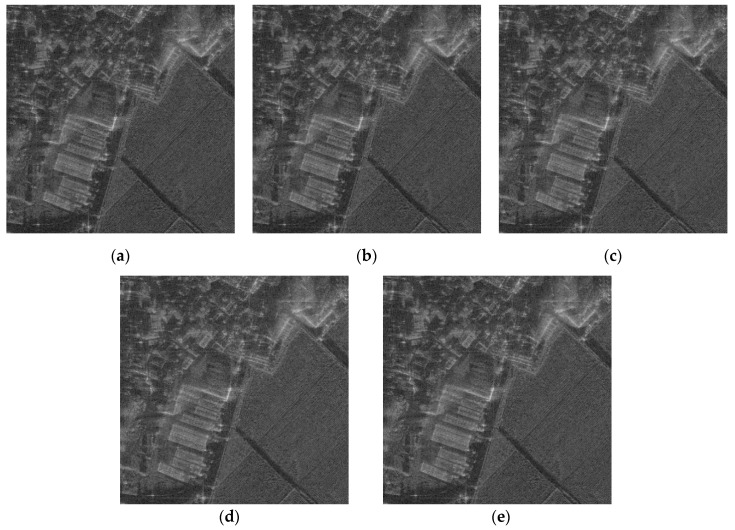
SAR image corrected by proposed FPA with the threshold set at (**a**) 0.01, (**b**) 0.1, (**c**) 0.3, (**d**) 0.9, (**e**) varying.

**Figure 5:**
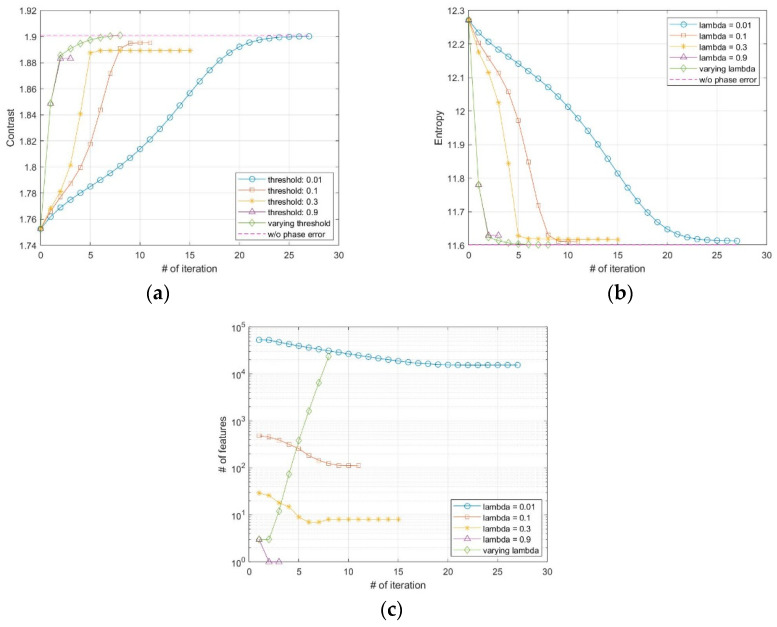
Variation of the quantitative measures of (**a**) contrast, (**b**) entropy, and (**c**) the number of features at each iteration.

The SAR image used for the proposed FPA is shown in Figure 2a. The size of the image is 4000 × 4000 pixels. The vertical and horizontal coordinates of the image represent the range and azimuth, respectively. The definitions of coordinates are the same for all of the other SAR images in this paper. Figure 2b,c shows the image enlarged at the point near the center of the image both without and with the phase error of Figure 3, respectively. The images corrected by FPA are shown in Figure 4, and the variation of contrast and entropy at each iteration are represented in Figure 5. The images in Figure 4a–d are the results for a constant threshold λ fixed by 0.01, 0.1, 0.3, and 0.9, respectively. Although the images look similar to each other, their contrast and entropy are slightly different, as shown in Figure 5 and Table 1. For a small value of λ, the convergence rate is low, even though the measures tend to converge to its optimal value. On the contrary, a large value of λ enables fast convergence, whereas the final performances are degraded. The reason for these characteristics can be explained by the number of features in Figure 5c. It shows that the smaller value of the threshold applied, the larger the number of features the algorithm uses for each iteration. As mentioned in the previous section, the features may contain the artifacts if the number of features is too large, which causes an adverse effect on the convergence rate. In the case of a small number of features, on the other hand, some dominant scatterers are omitted and it may interfere with the global optimality of the estimation, even if it achieves fast convergence. 

As explained in the previous section, we applied a varying threshold to the proposed FPA to satisfy both fast convergence and optimal performance. We set the threshold λ for initial iteration to a relatively large value, and gradually decrease the value at each iteration as in (23). The initial threshold $\lambda_{0}$ and the forgetting factor α are set to 0.9 and 0.5, respectively, and the corrected image from this method is shown in Figure 4e. As shown in Figure 5a,b, the quantitative measures reach the optimal value with an appropriate iteration number. Unlike the fixed threshold cases, the number of features increases for each iteration, as shown in Figure 5c, which enables the features of the current iteration to contain the dominant scatterers omitted at the previous iteration.

### 4.2. Comparison with Existing Autofocus Algorithms

We have compared the proposed method with existing postprocessing autofocus methods of PGA [5], GPGA [7], and ME [12]. Constant false alarm rate (CFAR) detection was used to select the strongest scatterers for GPGA, which is a modified algorithm of PGA. The stop conditions for PGA, GPGA, and ME were the same as that of proposed FPA, and we used the varying threshold for FPA as described in Section 3.3. The value of $\lambda_{0}$ and α for FPA were the same as the previous experiment.

**Figure 6:**
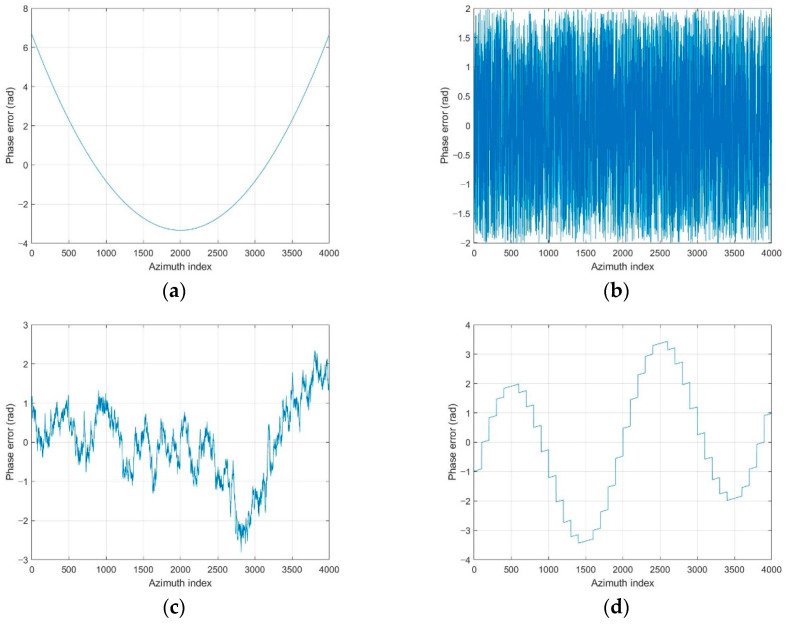
Phase errors to verify the performance of the autofocus algorithms. (**a**) Quadratic error. (**b**) Uniformly distributed random error. (**c**) Wiener process error. (**d**) Sinusoidal error with discontinuity.

**Figure 7:**
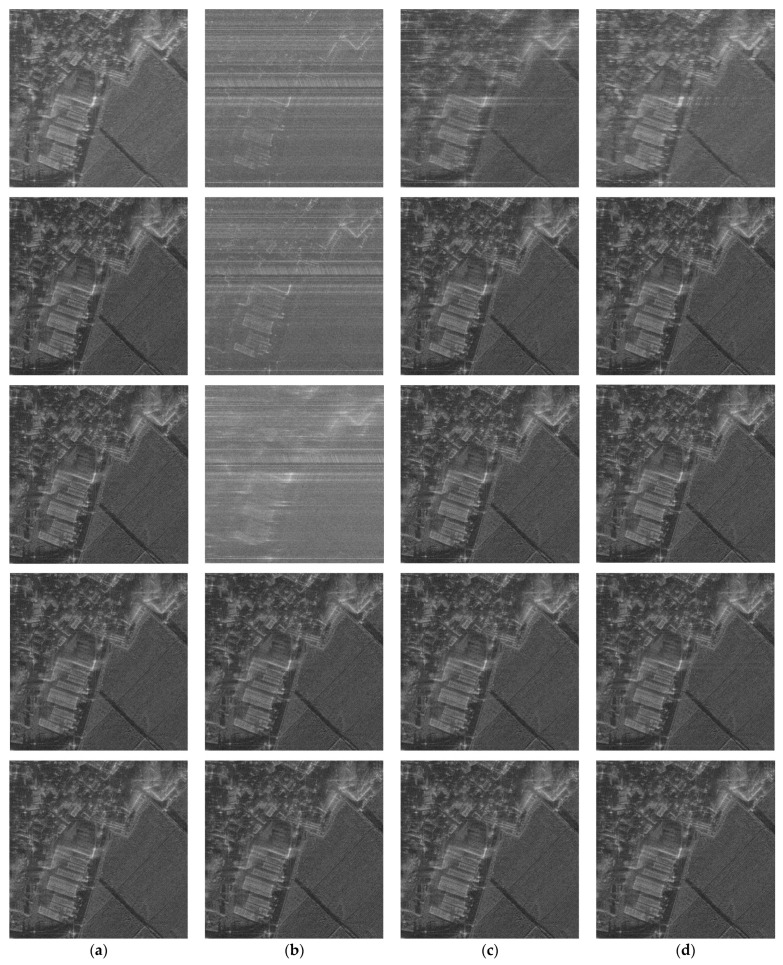
Phase-corrected images for different types of errors and autofocus algorithms: first row—without autofocus; second row—phase gradient autofocus (PGA); third row—generalized phase gradient autofocus (GPGA); fourth row—minimum entropy (ME); fifth row—proposed FPA. (**a**) Results for quadratic error. (**b**) Results for uniformly distributed random error. (**c**) Results for Wiener process noise. (**d**) Results for sinusoidal error with discontinuity.

**Figure 8:**
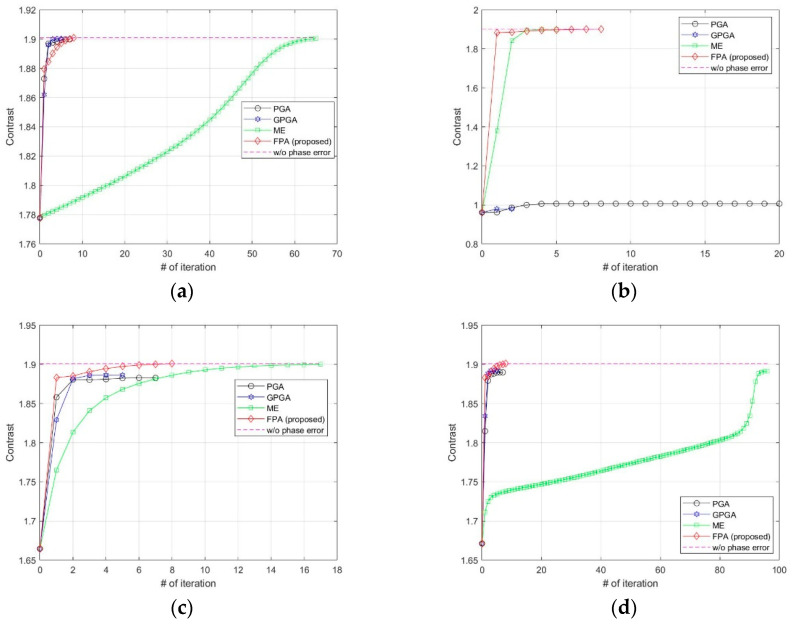
Contrast variations at each iteration for (**a**) quadratic error, (**b**) uniformly distributed random error, (**c**) Wiener process error, (**d**) sinusoidal error with discontinuity.

**Figure 9:**
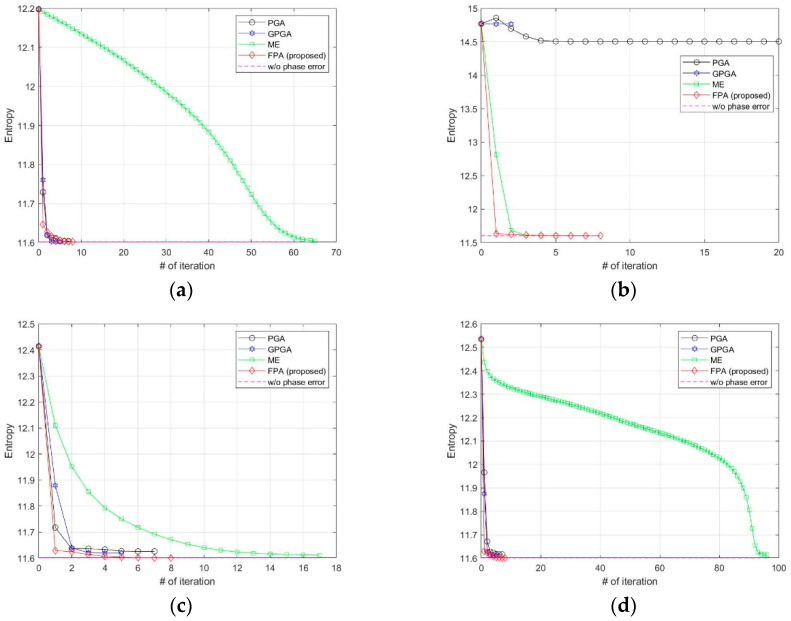
Entropy variations at each iteration for (**a**) quadratic error, (**b**) uniformly distributed random error, (**c**) Wiener process error, (**d**) sinusoidal error with discontinuity.

We added various types of phase errors as shown in Figure 6 to the scene in Figure 2a. The images corrected by PGA, GPGA, ME, and the proposed FPA are shown in Figure 7. The contrast and entropy variation for each iteration are represented in Figure 8 and Figure 9, respectively. The performance measures of the images for each method and phase error are represented in Table 2, and the number of iteration and total computing time are shown in Table 3. The computation time was measured with a workstation equipped with Intel^®^ Xeon^®^ Gold 6140 CPU. MATLAB was used as the programming language.

It is well-known fact that PGA shows fast convergence and sufficient performance for low-frequency errors, such as the quadratic error shown in Figure 6a, which can be observed from the results in Figure 7a, Figure 8a and Figure 9a, and Table 2 and Table 3. GPGA shows almost the same result with PGA with less iteration, but requires more computation time due to the additional process such as the strongest peak selection through CFAR for every iteration. The proposed method shows a similar trend with less computation time, because both PGA and GPGA require additional procedure for center-shifting, windowing, elimination of linear phase, etc. Meanwhile, ME requires more iterations due to the large phase error, even though it slowly converges to the optimal performance as shown in those figures. Unlike the quadratic error case, the image with random phase error cannot be corrected by PGA and GPGA, as shown in Figure 7b, Figure 8b and Figure 9b, and Table 2 and Table 3. It is a natural result because of the assumptions and limited window size of PGA, described in Section 1. Meanwhile, ME and FPA show nearly optimal performance with few iterations. Therefore, it can be inferred from these results that the performance and convergence rate of the proposed FPA are not limited by the bandwidth of the phase error, unlike PGA and ME.

**Figure 10:**
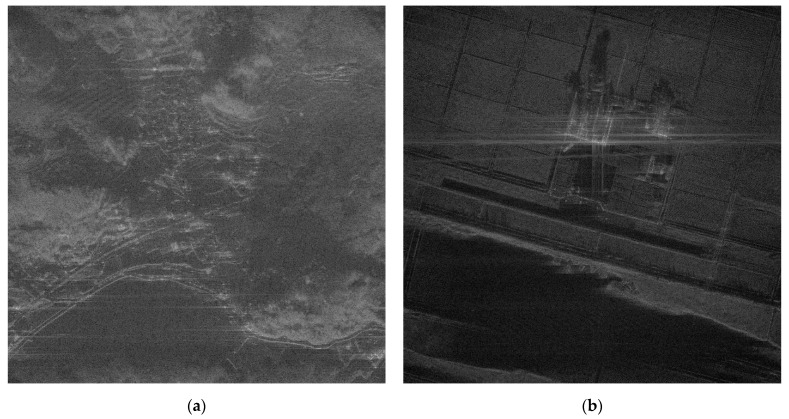
Additional scene to verify the reliability of FPA: (**a**) scene A and (**b**) scene B.

In a practical SAR system, Wiener process and discontinuous phase error can occurr because of the navigation systems for motion compensation. If we use an INS system, the Wiener process errors are generated due to the integration of IMU measurements that contain the Gaussian white noises. Furthermore, the navigation data experience some discontinuities if the system uses GPS measurement updates. The phase errors in Figure 6c,d are this kind of error, and the autofocus results for these errors are represented in (c) and (d) of Figure 7, Figure 8 and Figure 9, and Table 2 and Table 3. These results verify that FPA also shows the best performance with appropriate iteration number and computation time. Therefore, we verified the performance and convergence of the proposed FPA as well as its robustness for most types of phase errors through these results. We performed the same procedure for two more scenes in Figure 10 to verify the reliability of the algorithm. Scenes A and B are selected to have a higher and lower value of entropy, i.e., lower and higher contrast, than those of the image in Figure 2a. The results for these two scenes are represented in Table 4, Table 5, Table 6 and Table 7, and similar trends of performance and convergence are observed when compared to that of the results in Table 2 and Table 3. FPA shows best performance with sufficiently small iterations and computation time for all cases, and it verifies that FPA produces reliable performance.

## 5. Conclusions

In this paper, we proposed and demonstrated a new autofocus method for postprocessing of a phase-corrupted SAR image based on minimization of the cost function that consists of fidelity and regularization terms. The equation to achieve the optimality is derived by indirect optimization, and the algorithm to solve the equation is proposed. Each iteration in the proposed algorithm requires only one soft-thresholding for the reference image formation, and it enables more efficient processing than the existing regularization-based autofocus such as SDA. The tradeoff between the performance and convergence for the proposed FPA can be compromised by selecting proper constant threshold or using an asymptotically decreasing threshold with appropriate initial value and forgetting factor. The experimental results verified its better performance, convergence, and robustness when compared to the existing methods of PGA and ME. We verified the reliability of the proposed method by performing additional two experiments with a different scene.

Although the proposed FPA shows sufficient performance and convergence for these experiments, there are still remaining factors to improve, such as selection of features and determining the threshold for each iteration. These factors would depend on the scene. Hence, the modifications through adaptive methods would improve the performance and convergence of the proposed algorithm, which are the future works for this study.

## Figures and Tables

**Table 1:** Quality of the images with different conditions.

Conditions for Image Formation	Contrast	Entropy
Original image reconstructed by PFA	1.901	11.601
Phase-corrupted image	1.753	12.271
Image corrected by FPA with λ=0.01	1.900	11.613
Image corrected by FPA with λ=0.1	1.895	11.611
Image corrected by FPA with λ=0.3	1.889	11.617
Image corrected by FPA with λ=0.9	1.883	11.630
Image corrected by FPA with varying λ	1.901	11.601

**Table 2:** Comparison of performance for different autofocus algorithms.

Autofocus Algorithm	Contrast				Entropy			
	(a)	(b)	(c)	(d)	(a)	(b)	(c)	(d)
No autofocus	1.778	0.961	1.665	1.671	12.198	14.768	12.414	12.535
PGA	1.900	1.006	1.883	1.890	11.604	14.504	11.626	11.617
GPGA	1.900	0.979	1.886	1.892	11.602	14.764	11.620	11.612
ME	1.900	1.900	1.900	1.891	11.604	11.604	11.612	11.614
Proposed FPA	1.901	1.901	1.901	1.901	11.603	11.601	11.601	11.601

(a)–(d) denote the types of phase errors, same as Figure 6.

**Table 3:** Comparison of the number of iterations and computation time for different autofocus algorithms.

Autofocus Algorithm	Number of Iterations				Computation Time (s)			
	(a)	(b)	(c)	(d)	(a)	(b)	(c)	(d)
No autofocus	-	-	-	-	-	-	-	-
PGA	7	20	7	7	51.7	146.8	58.6	55.9
GPGA	5	2	5	5	77.0	37.0	89.9	84.5
ME	65	5	17	96	252.6	22.3	65.5	344.7
Proposed FPA	8	8	8	8	33.9	31.9	33.4	26.8

(a)–(d) denote the types of phase errors, same as Figure 6.

**Table 4:** Comparison of performance for different autofocus algorithms for scene A.

Autofocus Algorithm	Contrast				Entropy			
	(a)	(b)	(c)	(d)	(a)	(b)	(c)	(d)
No autofocus	1.590	0.873	1.519	1.544	12.965	15.209	13.164	13.194
PGA	1.616	0.893	1.614	1.619	12.715	15.269	12.710	12.702
GPGA	1.616	0.930	1.616	1.620	12.713	14.908	12.704	12.698
ME	1.615	1.626	1.624	1.618	12.726	12.690	12.699	12.821
Proposed FPA	1.624	1.626	1.626	1.626	12.707	12.687	12.687	12.687

(a)–(d) denote the types of phase errors, same as Figure 6.

**Table 5:** Comparison of the number of iterations and computation time for different autofocus algorithms for scene A.

Autofocus Algorithm	Number of Iterations				Computation Time (s)			
	(a)	(b)	(c)	(d)	(a)	(b)	(c)	(d)
No autofocus	-	-	-	-	-	-	-	-
PGA	6	3	7	7	49.8	24.9	59.3	61.0
GPGA	6	6	6	6	101.4	101.5	111.8	114.5
ME	83	12	23	99	325.9	52.4	109.9	465.0
Proposed FPA	10	9	9	9	35.7	39.4	40.6	46.2

(a)–(d) denote the types of phase errors, same as Figure 6.

**Table 6:** Comparison of performance for different autofocus algorithms for scene B.

Autofocus Algorithm	Contrast				Entropy			
	(a)	(b)	(c)	(d)	(a)	(b)	(c)	(d)
No autofocus	9.087	4.053	8.046	8.306	6.540	10.951	6.934	7.139
PGA	9.624	4.261	9.314	9.466	5.611	10.371	5.652	5.634
GPGA	9.622	4.333	9.349	9.485	5.612	10.175	5.646	5.631
ME	9.634	7.812	9.623	9.459	5.617	6.058	5.627	5.663
Proposed FPA	9.647	9.647	9.647	9.640	5.614	5.612	5.612	5.649

(a)–(d) denote the types of phase errors, same as Figure 6.

**Table 7:** Comparison of the number of iterations and computation time for different autofocus algorithms for scene B.

Autofocus Algorithm	Number of Iterations				Computation Time (s)			
	(a)	(b)	(c)	(d)	(a)	(b)	(c)	(d)
No autofocus	-	-	-	-	-	-	-	-
PGA	7	10	7	7	49.9	65.3	53.6	55.8
GPGA	6	5	7	7	108.3	97.1	110.2	118.8
ME	70	5	16	94	201.2	11.5	67.6	420.5
Proposed FPA	9	9	9	8	25.6	27.6	25.4	35.7

(a)–(d) denote the types of phase errors, same as Figure 6.

## Data Availability

Not applicable.
